# Conventional and Omics Approaches for Understanding the Abiotic Stress Response in Cereal Crops—An Updated Overview

**DOI:** 10.3390/plants11212852

**Published:** 2022-10-26

**Authors:** Kasinathan Rakkammal, Arumugam Priya, Subramani Pandian, Theivanayagam Maharajan, Periyasamy Rathinapriya, Lakkakula Satish, Stanislaus Antony Ceasar, Soo-In Sohn, Manikandan Ramesh

**Affiliations:** 1 Department of Biotechnology, Science Campus, Alagappa University, Karaikudi 630003, Tamil Nadu, India; 2 Department of Biological Sciences, North Carolina State University, Raleigh, NC 27606, USA; 3 Department of Agricultural Biotechnology, National Institute of Agricultural Sciences, Rural Development Administration, Jeonju 54874, Korea; 4 Department of Biosciences, Rajagiri College of Social Sciences, Cochin 683104, Kerala, India; 5 Applied Phycology and Biotechnology Division, Marine Algal Research Station, Mandapam Camp, CSIR—Central Salt and Marine Chemicals Research Institute, Bhavnagar 623519, Tamil Nadu, India

**Keywords:** abiotic stress, functional genomics, transcriptomics, proteomics, stress response

## Abstract

Cereals have evolved various tolerance mechanisms to cope with abiotic stress. Understanding the abiotic stress response mechanism of cereal crops at the molecular level offers a path to high-yielding and stress-tolerant cultivars to sustain food and nutritional security. In this regard, enormous progress has been made in the omics field in the areas of genomics, transcriptomics, and proteomics. Omics approaches generate a massive amount of data, and adequate advancements in computational tools have been achieved for effective analysis. The combination of integrated omics and bioinformatics approaches has been recognized as vital to generating insights into genome-wide stress-regulation mechanisms. In this review, we have described the self-driven drought, heat, and salt stress-responsive mechanisms that are highlighted by the integration of stress-manipulating components, including transcription factors, co-expressed genes, proteins, etc. This review also provides a comprehensive catalog of available online omics resources for cereal crops and their effective utilization. Thus, the details provided in the review will enable us to choose the appropriate tools and techniques to reduce the negative impacts and limit the failures in the intensive crop improvement study.

## 1. Introduction

The plant is a sessile organism that serves as the foundation for all living organisms on Earth and as a valuable resource for humans. All cereal crop species are members of the grass (Poaceae or Gramineae) family, the fourth largest family of flowering plants [1]. The major cereal crops, rice, maize, sorghum, finger millet, foxtail millet, wheat, barley, and other cereal crops, are the world’s primary food sources [2]. Food is necessary to provide energy for body growth, function, and defense. Our body’s basic requirements include proteins, minerals, carbohydrates, fiber, vitamins, and lipids, all essential nutrients. Among these requirements, carbohydrates, proteins, and fats which are needed in large quantities are called “macronutrients,” and the requirements of minerals and vitamins needed in small amounts are called “micronutrients” [3]. Cereals such as rice are enriched with carbohydrates; wheat is rich in carbohydrates and certain vitamins (B6); maize is rich in vitamins and micro elements, viz., P, Mg, Mn, Ca, Zn, Cu and Fe (depending on the varieties); millets are rich in fiber and proteins (https://data.nal.usda.gov/ (accessed on 16 October 2022)). These cereals are essential for the routine life of humans, and their production should be increased owing to the increasing population. However, abiotic stress is a global problem, limiting global crop production and reducing the yields of the plants by more than 50% in quality [4]. Abiotic stress conditions such as drought, high temperatures, salinity, mineral deficiency, and metal toxicity are usually experienced by plants in both natural and agricultural systems [5,6,7]. Among these stress factors, drought (low water availability), heat (extreme temperature), and salinity (high salt content) are the most important stresses, having a huge effect on the growth and productivity of the crops [8,9,10].

**Figure 1:**
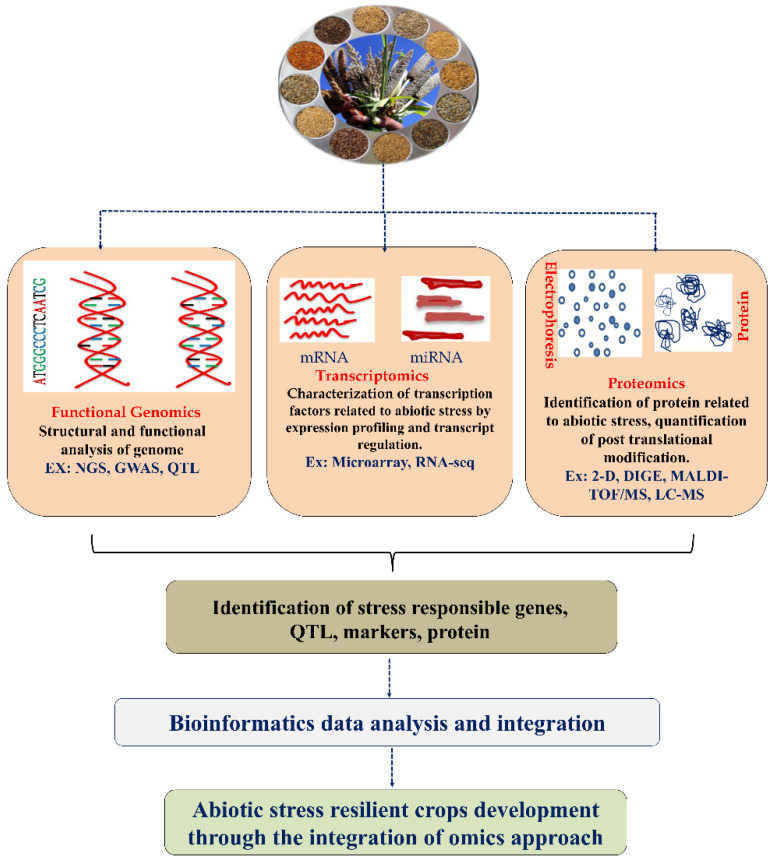
A schematic representation of omics approaches for identification of major regulator of stress response in the plant. QTL—quantitative trait locus; MAS—marker-assisted selection 2D gel electrophoresis; DIGE—differential gel electrophoresis; MALDI-TOF—matrix-assisted laser desorption/ionization time-of-flight; LC-MS/MS—liquid chromatography/mass spectrometry. Sources of the cereal image (https://agritech.tnau.ac.in/agriculture/millets_miracle_strategies.html (accessed on 17 October 2022)).

Furthermore, the world population is expected to reach nine billion by 2050, affecting average living standards. Food consumption also demands grain for livestock maintenance and agricultural land use, leading to the population’s food demand [8,11]. According to the FAO’s SOFI 2021 report (The State of Food Security and Nutrition in the World), the COVID-19 pandemic exposed agri-food systems to stress, resulting in increasing worldwide food instability and malnutrition [12]. There is an urgent need to increase crop productivity to meet the demand. Plants have developed sophisticated mechanisms to adapt to environmental changes and challenges that aid plant survival. The mechanism behind the environmental stress response in plants is undoubtedly more advanced and prominent than in animal cells [13]. Numerous fundamental reports are available on the molecular and cellular mechanisms behind abiotic stress adaptation in rice plants, which act as model plants for cereal crops. A plant’s response to drought, extreme temperature, and salinity depends on the regulation of genes (upregulation or downregulation). In this context, integrated omics research has been widely used to understand the plant’s biological networking and molecular mechanisms against various abiotic stresses. Despite tremendous progress in genomics, there is a need to study other omics levels, including transcriptomic and proteomic profiling, for a comprehensive understanding at the molecular level. Developing new crops that have improved resistance to various abiotic stress factors is essential. Plants are exposed to various abiotic stresses. Numerous stress-responsive genes are activated, which are involved in producing many proteins that help them activate and adjust the physiological and biochemical pathways in stress tolerance [14]. Therefore, integrating multi-omics data within the context of systems biology can provide more profound knowledge for future directions (molecular biology, genome editing, etc.) [15,16,17]. In this review, we discussed the recent advancements in functional genomics, transcriptomic, and proteomic analyses to understand the adaptation and tolerance mechanisms enhancing drought, extreme temperature, and salinity in different cereal crops. A summary of transcriptomic, proteomic, and functional genomic approaches for crop improvement is presented (Figure 1).

## 2. General Effects of Drought, Heat, and Salt Stress on Plant Growth and Development

**Figure 2:**
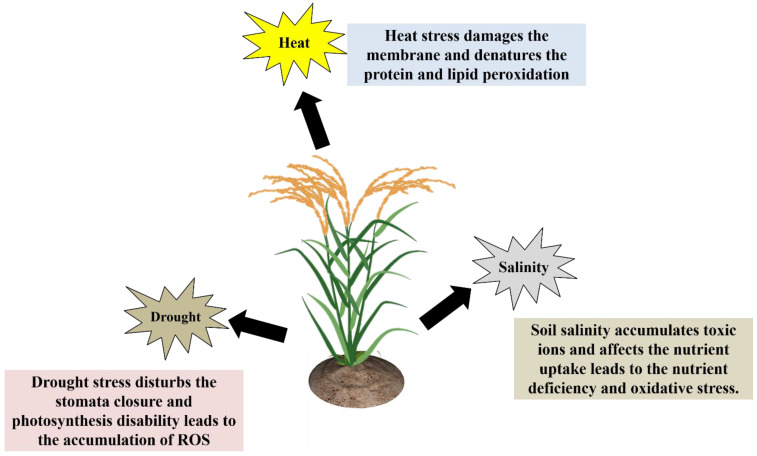
How do plants react to environmental stress factors?

Plants are multicellular, and their reactions to abiotic stresses are highly complicated and have passive-aggressive behavior. Depending on the environmental conditions, plants suffer from abiotic stresses that are either reversible or irreversible [14,17,18]. Besides that, how do plants react to environmental stress factors? Plant biologists and agronomists consider this to be an extremely severe problem due to the fact that it poses a significant risk to crop productivity. Plants have developed several biochemical, physiological, and metabolic responses to withstand environmental stress factors. Understanding plants’ molecular, cellular, physiological, and biochemical changes during abiotic stresses is crucial for better crop management [16,19,20]. Plants have primary and secondary stress response mechanisms to protect themselves from various abiotic stresses. The drought stress response involves the control of ion homeostasis (activation/inactivation of aquaporins), and water transport. Another general plant stress response is the synthesis of protective molecules or osmolytes, such as sugars, proline, polyalcohols, quaternary ammonium compounds, and so on, as well as different specific proteins such as heat shock proteins, LEA proteins, osmotic, etc., [16,19,20]. In the secondary stress response, plants generate “reactive oxygen species” (ROS), which includes H_2_O_2_ peroxidation and lipid peroxidation (MDA content was increased). Reduced glutathione, superoxide dismutase, catalase, ascorbate peroxidase, glutathione peroxidase, and other antioxidant enzymes are activated when ROS accumulates in the cell cytoplasm [21]. The general effects of drought, heat and salt stress on plant growth and development have been shown (Figure 2).

### 2.1. Drought Stress

The stress response of plants varies with each species, determined by the growth stage and environmental factors [22]. Drought stress reduces the yield of maize (63–87%) [23], wheat (57%) [24], and rice (53–92%) [25]. The underlying impact of drought is poor germination, damaged seedling development, decreased root and shoot dry weight, hypocotyl length, and vegetative development and these factors have been accounted for in imperative nutrition crops including rice, wheat, sorghum, foxtail millet, and finger millet, etc., [26,27,28,29,30]. Water and nutrient associations are important factors for the growth and development of plants. Stomatal conductance, leaf water potential, transpiration rate, and water relations are activated. Drought stress highly affects the above factors and the nutrient relationships such as N, Si, Mg, and Ca [31]. Furthermore, drought stress decreases the soil moisture and leads to a reduction in root growth, which is deeper and thicker. In this context, roots are the first organ that senses water availability and sends signals to the aerial organs via root-to-shoot xylem channels to decrease the turgidity of guard cells and close the stomatal aperture to reduce water loss [22,32]. Drought-responsive genes were divided into two categories: functional and regulatory genes. Synthase genes, aquaporin genes, protective metabolites (sucrose, proline, and betaine), and proteins (LEA protein, molecular chaperone) are all involved in cell defense against environmental stress. Stress-related transcription factor genes, protein kinase genes, phospholipid metabolism-related genes, and protein phosphatase genes are regulatory gene products that indirectly protect plant cells from abiotic stress [16,33,34,35].

### 2.2. Temperature Stress (Heat)

Temperature is a major factor affecting plants’ distribution, growth, and development. Plant morphological and physiological processes are affected by the temperature conditions under which the plant species grow, because each species grows in a specific temperature range (i.e., 25–30 °C for maximum growth) [36]. Elevated temperatures cause adverse effects such as the burning of shoots and leaves, leading to leaf senescence and growth inhibition [22,37]. Under this condition, plants try to balance their tissue water and moisture content to protect themselves from high temperatures. It can also reduce the germination, development of spikes, number of florets, and net assimilation rate in sorghum, rice, and maize [22,37]. Heat stress independently alters the physiology and metabolism of plants. However, its effect becomes enhanced when combined with other abiotic stresses such as drought and salt stress [22,38]. Heat stress in the reproductive stage causes a major reduction in the yield of crops due to the poor photosynthetic process. Extreme temperature stress leads to the deactivation of various enzymes involved in photosynthesis. For example, PSII enzymes are an important factor in photosynthesis; high temperatures influence the activity of PSII. Under high temperatures, components of PSII were damaged in wheat and barley [38,39]. Although high-temperature stress reduces the enzyme activities of adenosine diphosphate-glucose pyrophosphorylase, sucrose phosphate synthase, and invertase, it affects the starch and sucrose synthesis of plants [40,41].

### 2.3. Salinity Stress

Salinity is an abiotic stress that severely affects plant growth, development, and crop production. The response of plants under salinity stress can be described in two phases: the initial phase, an ion-independent response, which takes minutes to days to cause toxicity, affects stomatal closure and inhibits cell expansion, particularly in the shoot [22,42]. The second phase, the ion-dependent response, which takes days or weeks to build up cytotoxicity, causes premature senescence of leaves, reducing yield or even causing death [42,43,44]. Moreover, salt may affect plant growth indirectly by decreasing the rate of photosynthesis and stomatal conductance [45,46]. Stomata are the main structures responsible for gas exchange control. Salt stress reduces stomatal conductance by affecting their opening, size, and density [47]. Consequently, transpiration (i.e., water loss) and photosynthesis (CO_2_ uptake) rates are also reduced [48,49]. Increased Na^+^ ion is sensed by plants, leading to immediate closure of stomata and inhibition of leaf expansion within minutes of exposure. Later, due to excessive ion concentration, premature senescence of leaves and a reduction in yield occur in plants [47]. Compared to other cereals, rice is the most salt-sensitive crop. Chlorophyll and carotenoid contents in rice leaves were significantly decreased after the introduction of salt stress [50]. An excess amount of salt adversely affects the metabolic activities of plants, including cell wall damage, accumulation of electron-dense proteinaceous particles, plasmolysis, cytoplasmic lysis, and damage to ER. In addition, it accumulates citrate, malate, and inositol in leaf blades within one day of salt treatment [51]. Sorghum is a moderate, salt-tolerant agronomic crop. Swami et al. [52] reported that sorghum plants under salt-stress conditions reduced the growth of leaves and chlorosis. In sweet sorghum, salinity decreased germination percentage and increased germination duration [53,54].

### 2.4. Oxidative Damage and Antioxidant Enzyme Defense System in Plants under Abiotic Stress

Abiotic stresses affect plant growth, development, and productivity by degrading cellular metabolism and increasing reactive oxygen species (ROS) generation. During abiotic stress, photorespiration, the photosynthetic system, and mitochondrial respiration pathways contribute to the generation of ROS [55,56]. It has been proven that ROS are generated in different cellular compartments such as mitochondria, chloroplasts, peroxisomes, cytoplasm, and the extracellular region [17,57,58,59]. The increased production of ROS during stress can be damaging to cells. Still, reactive oxygen intermediate (ROI) acts as a signal to activate stress-response and defense pathways [60]. Thus, ROS can be considered a cellular indicator and secondary messenger in the stress-response signal transduction pathway. Approximately 1–2% of total molecular oxygen consumption results in the production of ROS in normal conditions. The stressed plant cell generates toxic ROS such as hydrogen peroxide (H_2_O_2_), singlet oxygen (^1^O_2_), alkoxyl (RO^•^), peroxyl (ROO^•^), hydroxyl radical (OH^•^), and superoxide (O_2_^•−^)[17,57,59,61,62]. Increased ROS levels can potentially cause peroxidation of lipids, denaturation of proteins, mutation of DNA, and cellular oxidative damage [59,63]. The equilibrium between ROS production and elimination at the intracellular level must be regulated to overcome ROS induced damage, and maintain the growth, metabolism, development, and overall productivity of the crops. Plants cells produce a complex of enzymatic and nonenzymatic antioxidants to maintain ROS homeostasis. The enzymatic antioxidant system includes several antioxidant enzymes such as superoxide dismutase (SOD), catalase (CAT), ascorbate peroxidase (APX), glutathione reductase (GR), peroxiredoxins (Prxs), and enzymes of the ascorbate-glutathione (AsAGSH) cycle, such as ascorbate peroxidase (APX), monodehydroascorbate reductase (MDHAR), dehydroascorbate reductase (DHAR). Nonenzymatic antioxidants such as glutathione (GSH), ascorbate (AsA), tocopherol, carotenoids, and phenolic compounds are also involved in the removal of ROS [64]. According to several reports, antioxidant enzyme activity is involved in the stress tolerance of plants. Alterations in SOD, CAT, APX, and GR activity were observed in various cereal crops under abiotic stress [65]. 

Superoxide dismutase is the most effective intracellular metalloenzyme that serves as the first line of defense against ROS-mediated oxidative stress. According to the metal cofactors, SODs are classified into three types: Cu/Zn-SOD (copper/zinc cofactor), Mn-SOD (manganese cofactor), and Fe-SOD (iron cofactor) [66,67,68,69]. SOD catalyzes the dismutation of the superoxide radicals (O_2_.^−^) into hydrogen peroxide (H_2_O_2_) and oxygen (O_2_) and decreases the formation of hydroxyl radical (OH^•^) formation via the metal-catalyzed Haber–Weiss-type reaction. The dismutation rate is 10,000-fold higher than spontaneous dismutation [59]. Under stressful environments, upregulation of SOD has been associated with plant survival and mitigation of oxidative damage. A significant increase in SOD activity under drought stress has been observed in many plant species, viz., rice [70], wheat [71], sorghum, and sunflower [72]. 

Catalase is a tetrameric heme-containing enzyme involved in degrading H_2_O_2_ into H_2_O and O_2_. Under normal environmental conditions, catalase scavenges H_2_O_2_ produced during photorespiratory oxidation, mitochondrial electron transport, and β-oxidation of the fatty acids. Catalase enzyme is found in the mitochondria, peroxisomes, and cytoplasm of higher plants [73]. SOD detoxifies superoxide radicals (O_2_.^−^) into hydrogen peroxide (H_2_O_2_) and oxygen (O_2_), then then the H_2_O_2_ can be eliminated by CAT. 

In plant cells, AsA-dependent APX (EC 1.11.1.1) occurs in different isoforms (cytosolic APX (cAPX), mitochondrial APX (mtAPX), chloroplastic APX (chlAPX) and peroxisomal/glyoxysomal APX (mAPX). APX is the only enzyme capable of scavenging H_2_O_2_ in the chloroplast since CAT is not present, which participate in the AsA-GSH cycle producing monodehydroascorbate (MDHA) [74]. 

GR is a flavoprotein oxidoreductase involved in the defense system by sustaining the status of glutathione (GSH), a disulfide reductant, which protects thiol groups of enzymes, regenerates ascorbate, and reacts with singlet oxygen (^1^O_2_) and hydroxyl radical (OH^•^). GSH and GR play a key role in determining the tolerance of a plant under various stresses. This might be due to maintaining a high ratio of NADP^+^/NADPH, therefore ensuring the availability of NADP^+ ^ for accepting electrons from the photosynthetic electron transport chain and facilitating the regeneration of oxidized ascorbate [17,60,65].

## 3. Functional Genomic Approaches to Identify Stress-Responsive Genes in Cereals 

The genomics strategies help develop climate-resilient crop varieties to ensure food security by helping conserve genomic resources. The discovery of genomic variations and genes related to climate adaptation found in wild relatives of crop plants through whole-genome sequencing is an indicator of the development of environmentally-adapted crops [75,76]. Genomics and systems biology approaches toward the discovery of stress-tolerant traits have been made possible by the availability of molecular markers, QTL mapping, genetic mapping, and comparative genomics, the complex relationship of plants and environmental factors, and expression quantitative trait loci (eQTL). These techniques can provide a practical approach to identifying candidate genes involved in abiotic stress tolerance. 

### 3.1. Genome Sequencing

**Figure 3:**
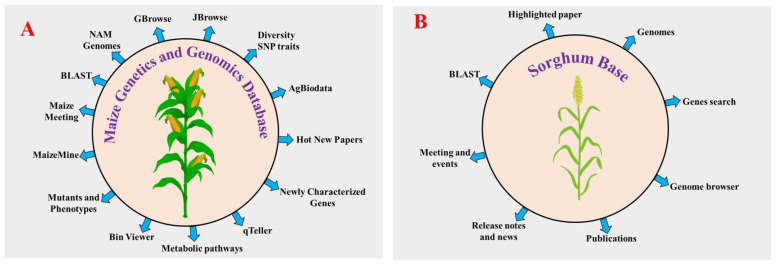
Graphical illustration of the maize genetics and genomics database (MaizeGDB) and sorghum base. Various tools have been implemented in the MaizeGDB (**A**) (https://www.maizegdb.org (accessed on 10 October 2022)) and sorghum base (**B**) (https://www.sorghumbase.org (accessed on 18 October 2022)), both of which help to collect all molecular data from the database.

Rice was the first cereal to be sequenced, which paved the way for NGS characterization of more complex cereals [77,78,79]. Afterward, most commonly available cereals had their genomes sequenced at the whole genome level, including maize [80], sorghum [81], foxtail millet [82,83], wheat [84], barley [85], finger millet [86], pearl millet, kodo millet, barnyard millet and green foxtail. Detailed information on published genome sequences of cereal crops is listed in Table 1. Over the past years, advancements have been made in DNA sequencing technology, facilitating the generation of a large amount of sequencing data within a short time in a cost-effective manner compared with the first-generation sequencing methods (Sanger-sequencing). Next-generation sequencing (NGS) technologies have high-throughput sequencing with prominent next-generation sequencing (NGS) platforms, including the Illumina/Solexa AB SOLiD Genome analyzer (https://www.illumina.com/ (accessed on 18 January 2022)) and Roche 454 GS FLX Titanium (www.454.com (accessed on 20 January 2022)). These technologies increase the eagerness for genome sequencing. First- and second-generation sequencing technologies have some disadvantages, so the researchers developed third-generation sequencing technologies to overcome certain drawbacks of first- and second-generation sequencing methods. In whole-genome sequencing of plants, single molecule sensor (HeliScope™) [87], single molecule real time sequencer (SMRT™) [88,89], single molecule real time sequencer (RNAP™) [90], and Nanopore DNA sequencers are used. They illustrate detailed genomic features including coding and noncoding genes, GC content, and repetitive and regulatory sequences that can be used to understand the functional roles of plant genes [91,92]. The NGS technology has opened the door to studying plant genomics to understand the abiotic stress response and to also produce improved crop varieties against abiotic stress. The study of plant species with larger genome sizes is more complicated due to the presence of repetitive elements in their genomes. These complex genomes are one of the most challenging problems for sequence assembly. For example, the genome size of the monocot plant wheat (hexaploid) is highly complex, consisting of 17 Gb, compared with the 4.79 Gb barley genome (diploid) [93,94]. Among the cereals, genome databases are available for rice and maize. Four other data genome databases are available for rice such as Rice Genome Annotation Project (RGAP) (http://rice.uga.edu/ (accessed on 25 January 2022)), International Rice Informatics Consortium (IRRI) (http://iric.irri.org/resources/rice-databases (accessed on 2 February 2022)), *O. sativa* genome database (OsGDB) (https://www.plantgdb.org/OsGDB/ (accessed on 12 February 2022)) and Rice RelativesGD (http://ibi.zju.edu.cn/ricerelativesgd/ (accessed on 17 February 2022)) [95,96,97]. All these databases contain annotated genomes of rice, and molecular resources (cDNA full length, gene, EST, markers, and expression data). The first genome sequence of maize was released in 2009 by Schnable et al. [80]. Researchers released several genome assemblies of maize from different countries afterwards. The maize genetics and genomics database (MaizeGDB) is the community database and global web source for maize [98,99,100]. More the 40 genome assemblies of maize inbred lines are available in addition to the B73 reference genome assembly. To date, MaizeGDB contains the five B73 reference genome assemblies of maize. The current B73 assembly version, Zm-B73-REFERENCE-NAM-5.0 (also known as RefGen_v5), released in January 2020, was sequenced and assembled along with a set of 25 inbred known as the Nested Associated Mapping (NAM) founder lines by the NAM Consortium using PacBio long reads and mate-pair strategy. The RefGen_v5 assembly sequence includes all 10 chromosomes. The first three assemblies (RefGen_V1, V2, and V3) were based on a bacterial artificial chromosome (BAC) sequencing strategy. The RefGen_V4 assembly used a new approach that relied on PacBio SMRT sequencing at Cold Spring Harbor to a depth of 60x coverage with scaffolds created via the assistance of whole genome restriction mapping (aka Optical Mapping). Maize-GDB also contains 38 additional reference genome assemblies, such as eight inbred lines (A188, B104, CMC247, Mo17, PH207, and W22), four European flints (DK105, EP1, F7, and PE0075), 25 NAM population (B97, CML52, 69, 103, 228, 247, 277, 322, 333, HP301, Il14H, Ki3, Ki11, Ky21, M37W, M162W, Mo18W and Ms71) and one teosinte (PI566673). Around 60 % of genes are found in all the NAM lines. Researchers have extensively used NAM populations to study maize flowering time, leaf structure, disease resistance, and other important agronomic traits. MaizeGDB hosts a wide range of data, including genome metadata, microRNAs, QTLs, SNPs, genome assemblies, genetic maps, ESTs, pathway da-ta, microarray data, RNA-seq, proteins, gene models, and transcripts. Several tools such as qTeller, maizemine, metabolic pathways, maize meeting, bin viewer, newly characterized genes, diversity SNP traits, and AgBioData have been implemented to improve the accessibility and visualization of data (Figure 3) [101]. Among these, qTeller is a comparative RNA-seq expression platform that helps to compare expression between two genes visually. Maizemine is a data mining respiratory for the MaizeGDB [102]. It enables researchers to create and export customized annotated datasets that can be combined with their research data for use in downstream analyses. Using the maize meeting tool, researchers can find past, present, and future information about maize genetics meetings. These recent updates at MaizeGDB will serve as a template for other databases to manage large-scale pan-genomes of any species. More than 15 genome assemblies are currently available at the SorghumBase database (https://sorghumbase.org/ (accessed on 18 October 2022)). As with rice, sorghum and maize, the development of genomic databases for other cereals will enable researchers to gather information about all molecular data.

Among the small millets, foxtail millet genome sequence has been sequenced by two research institutes (US Department of Energy Joint Genome Institute and Beijing Genomics Institute) [83,103]. The genome size of foxtail millet is around 515 Mb. A total of 38,801 genes were predicted (30,579 genes annotated and 8220 genes unannotated) from the genome sequence of foxtail millet. Bennetzen et al. [83] predicted more than 25,000 protein-encoding genes and 63,286 expressed sequence tags (ESTs) from their foxtail millet genome sequence. Like foxtail millet, two research groups released finger millet’s draft genome sequence in 2017 and 2018 [86,104]. The Hittalmani et al. [86] finger millet draft genome consisted of 525,759 scaffolds (>200 bp) with N50 length of 23.73 Kb, and the average scaffold length of 2275 bp. They have predicted 78,647 non-transposable elements and 6596 transposable elements-related genes from their draft genome sequences of finger millet using a de novo gene prediction method using Augustus. Furthermore, they have also predicted drought stress-responsive genes (2866), calcium transport and accumulation genes (330), and C_4_ pathway genes (146) from the same draft genome sequence of finger millet. The released draft genome sequences of finger millet are publicly available at the National Center for Biotechnology Information (NCBI) (Bio-Sample numbers: SAMD00076255 and SAMN04849255). The School of Plant Sciences, Ecology and Evolutionary Biology, Arizona Genomics Institute recently released a thoroughly annotated genome sequence (version 1.0 assembly) at the Phytozome database (https://phytozome-next.jgi.doe.gov/info/Ecoracana_v1_1 (accessed on 4 October 2022)). The genome assembly was generated by a MECAT assembler [105] and subsequently polished using QUIVER. The released genome assembly (version 1.0) contains a 1110.3 Mb sequence, consisting of 674 contigs. In 2018, the China Agricultural University submitted the shotgun genome sequence of proso millet (923 Mb) at NCBI (bio-sample and bio-project numbers are SAMN08335224 and PRJNA429322, respectively) [106]. Around 55,930, 339, 1420, 1640 and 2302 protein-coding genes, microRNAs, transfer RNAs, ribosomal RNAs and small nuclear RNAs were identified from the draft genome sequences of proso millet, respectively. The draft genome sequence for barnyard millet was generated by [107]. The genome size of barnyard millet was estimated to be 1.27 Gb with a scaffold N50 length of 1.8 Mb. About 917 *cytochrome P450 monooxygenase*, 277 *glutathione S-transferase*, 4945 differentially expressed genes, 108,771 protein-coding genes, 785 microRNAs and other non-coding RNAs were predicted from the draft genome sequence of barnyard millet. The International Crops Research Institute for the Semi-Arid Tropics (ICRISAT), Hyderabad, India, has released the draft genome sequence of pearl millet (~1.79 Gb) [108]. They used whole genome shotgun and bacterial artificial chromosome sequencing techniques to assemble the pearl millet genome. About 27,893 (72.30%) genes were annotated and 10,686 (27.70%) genes unannotated. The annotated and drafted genome sequences of all cereals will help to identify the genes and markers and will help to improve the growth and production of cereals under both biotic and abiotic stresses. With the enormous amount of data obtained from these sequencing methods, the major question is how to analyze and utilize this information. This has prompted scientists to develop bioinformatics tools capable of extracting biologically meaningful information from a large amount of data. The genome assemblers are TIGR assembler [109], CAP3 [102], string graph assembly [110], Newbler [111], SSAKE [112], VCAKE [113], SHARCGS [114], ALLPATHS-LG [115], Edena [116], Velvet [117], CABOG [118], ABySS [119], Genomic Analysis Toolkit [120], and SOAPdenovo2 [121]. They have been developed to reconstruct the whole genome sequence by aligning and merging sequence reads generated from the current genome sequencing technologies. CAP3 is the third generation of contig assembly programs. It helps to compute the assembly of large genomes such as mice, humans and maize [122]. CAP3 has been successfully used for the EST assembly of several plant species including, rice, wheat, and maize [123,124]. The quantitative trait loci sequencing (QTL-seq) strategies can be used to conquer the challenges of complex genomes. They can be used to detect QTL in large populations [125].

**Table 1:** List of important cereal crops sequenced.

S. No.	Crop (Common Name)	Botanical Name	Genome Size (Mb)	Sequencing Methods	Reference
1	Rice	*Oryza sativa* ssp. *japonica* (Nipponbare)	420	Sanger, WGS	[78]
		*Oryza sativa* ssp. *japonica* (Nipponbare)	389	Sanger, BAC-by-BAC	[79]
		*Oryza sativa* ssp. *indica*	466	Sanger, WGS	[77]
2	Maize	*Zea mays (Palomero Toluqueno)* (popcorn)	2100	Sanger, WGS	[126]
		*Zea mays (B73)*	2300	Sanger, BAC-by-BAC	[80]
3	Sorghum	*Sorghum bicolor* (L.) *Moench*	730	Sanger, WGS	[81]
4	Foxtail millet	*Setaria italica*	515	Illumina, WGS	[82]
5	Bread wheat	*Triticum aestivum*	17,000	454, WGS	[84]
6	Barley	Hordeum vulgare	5100	454, BAC-by-BAC	[85]
7	Finger millet	Eleusine coracana	1200	Illumina, WGS	[86,104]
8	Pearl millet	*Cenchrus americanus*	1800	Illumina, WGS	[108]
9	Proso millet	*Panicum miliaceum*	923	Illumina short-read coupled with Pac-Bio long-read sequencing	[106]
10	Barnyard millet	*Echinochloa esculenta*	1.27	Illumina HiSeq platform	[107]

### 3.2. Molecular Markers

The availability of genome sequence data has provided new potential resources for cereal crop improvement. Still, the genome sequence’s large size and non-coding parts were bottlenecks associated with the genetic information. It is not easy to sequence and obtain information about the genes from proteomics. Expression sequence tags (EST) were developed to overcome the large and non-coding genome problems. With this method, a cDNA library can be produced from different tissues at different developmental stages and under different stress conditions, enabling the identification of transcripts related to tissues or stress-specific conditions. It is easy to obtain complicated and targeted plant species sequencing under abiotic stress conditions. It also provides the mRNA with a sufficient expression profile of specific sequences from cDNA under stressed conditions. EST data were generated and made available (http://www.ncbi.nlm.nih.gov/dbEST (accessed on 5 March 2022)). The NCBI-EST database contains approximately 449,101 ESTs for drought, 312,353 ESTs for salinity, 103,898 ESTs for low temperature, and 252,595 ESTs for high temperature [127,128]. PlantGDB (http://www.plantgdb.org/ (accessed on 5 October 2022)) provides genome browsers to display current gene structure models and transcript evidence from spliced alignments of EST and cDNA sequences. CerealESTdb comprises ESTs from four major cereal crops, namely rice (*Oryza sativa* L.), wheat (*Triticum aestivum* L.), sorghum (*Sorghum bicolour* L.), and maize (*Zea mays* L.), under various abiotic stress, viz., salt, drought, heat, cold and ABA. This database consists of 55,826 assembled EST sequences, 51,791 predicted genes models, and their 254,609 gene ontology terms including extensive information on 1746 associated metabolic pathways. The CerealESTdb is publicly available with the URL http://cabgrid.res.in/CerealESTDb (accessed on 5 October 2022) [129].

Molecular markers are the most important tools to develop and improve selection efficiency in identifying novel agronomic traits. Molecular markers are used for phylogeny and evolution studies, analysis of exotic germplasm diversity, cultivar genotyping, biotic and abiotic stress resistance, etc. [130]. A large number of molecular markers, such as SSRs (simple sequence repeats) and SNPs (single-nucleotide polymorphisms), have provided new strategies for germplasm characterization and genetic improvement, including breeding for resistance to abiotic stresses [131]. SSRs (simple sequence repeats) are 1–6 nucleotides contain multi-allelic and co-dominant at a single locus. The tandem arrays of SSR motifs mutate at the rate of 10–7 to 10–3 mutations per locus/generation [132]. Therefore, the number of repeat units in an individual genotype leads to high polymorphic and genotypic variation, making these sites useful for genetic analysis and breeding. A total of more than 20,000 SSR primers for rice have been developed [133]. McCouch et al. [134] developed 2240 di, tri, and tetra nucleotide markers and experimentally validated them. Among these, 56 SSR markers of *O. sativa* match with the BAC clone of the *O. sativa* genome. In rice, QTL-linked SSR markers generated 99 polymorphic alleles in 142 rice genotypes [135]. Based on the PIC (polymorphism information content) value (0.991), marker RM8094 proved to be an appropriate marker to distinguish a salt-tolerant rice variety from a salt-sensitive variety [135]. Similarly, RM104 is a marker to differentiate drought tolerance in rice. It is evident that the genomic region RM212–RM302–RM3825 on chromosome 1 is connected to drought resistance traits and can be beneficial in marker-assisted breeding for drought resistance in rice [136].

In recent years, SNP-based diversity analysis has been used in several plants [131,137]. It can dominate other molecular markers because of their irregular occurrence and minimal level of mutation [138]. The NCBI SNP network databases (https://www.ncbi.nlm.nih.gov/projects/SNP/ (accessed on 10 March 2022)) allow us to identify functional SNPs. CerealsDB (https://www.cerealsdb.uk.net/cerealgenomics/CerealsDB/indexNEW.php (accessed on 5 October 2022)) was created by members of the Functional Genomics Group at the University of Bristol. It provides information about the SNP in the genomes of bread wheat (*Triticum aestivum*) and its relatives [139]. Various software is readily available to visualize, analyze, and identify SNPs, including SNPsniffer, SeqMan genome analyzer, Atlas-SNP, ssahaSNP, PanGEA, CLCbio, MAQ, and NextGENe [91,140]. SNPs have been used to identify QTLs and ESTs associated with drought, salinity, and heat stress. In rice, the regulatory region of ERF3 employed to detect functional SNPs in drought stress tolerance revealed 31 SNPs and short InDels [141]. TaDREB1 is an important transcription factor present in wheat plants, which is involved in drought stress. Chen et al. [142] identified 271 SNPs and 14 InDels of TaDREB1 transcription factor associated with drought stress. SNP marker analysis between the heat-tolerant (K7903) and heat-susceptible (RAJ4014) genotypes of heat shock protein HSP16.9 revealed the SNP (A/G) at the thirty-first amino acid position, resulting in a point mutation from Asp to Asn [143]. In *Zea mays*, association mapping with candidate genes leads to identifying SNPs associated with modifying abscisic acid levels in floral tissue during drought conditions [144]. Zea mays cultivars from Serbia and Bulgaria were used to identify the SNPs of a MYBE1 transcription factor. Only the Ser/Thr-rich region of MYBE1 is affected by SNP mutation, not the conserved R1 domain. The detected SNP mutations are associated with genes related to drought stress tolerance in maize [145]. The SNP linkage map of 50 barley genotypes in response to terminal drought stress during plant growth and development and the expression patterns of drought-regulated genes resulted in the identification of 17 starch synthesis/degradation genes [146,147,148]. 

Comparative plant genomics is a crucial technique to examine the similarities and variations in genomes between plant species. Comparative genome mapping enables us to understand the patterns and strategies that regulate plant genome evolution and uncover functional regions of genomes [149]. Plant genomes are different due to insertions, deletions, duplications, inversions, and translocations, but notable similarity occurs in the genomes of different species [150]. These alterations play an important role in genetic and phenotypic diversity studies. The genomes of monocot plants are highly conserved compared to dicot plants [151]. Therefore, there is a possibility to illuminate the genetic information of monocot plants. Several mapping studies quantify the intraspecific genome variation and aid in identifying numerous QTLs associated with salinity, drought, and extreme temperatures [127,149]. QTL mapping is an efficient method compared to previously listed DNA markers such as RFLP, AFLP, RAPD, SSR, and SNP [152]. The major cereal crops are mapped through QTL, available at the Gramene database (http://www.gramene.org/qtl/ (accessed on 5 October 2022)). It contains QTLs related to abiotic stresses such as drought, salinity, cold, extreme temperature, nutrient deficiencies, and heavy metal toxicity. The Sorghum QTL Atlas (https://aussorgm.org.au/sorghum-qtl-atlas/ (accessed on 6 October 2022)) consists of 150 QTL and GWAS studies that have been published in sorghum from 1995 to present. It is helpful for the comparative genomics and crop improvement of sorghum [153]. WheatQTLdb V2.0 (http://www.wheatqtldb.net/ (accessed on 6 October 2022)) QTL database for wheat that includes information about tolerance to abiotic stresses including drought, water logging, heat, pre-harvest sprouting, salinity, biotic stresses, biofortification (Fe/Se/Zn contents), morphological and physiological traits. This web resource has a collection of QTLs (27,518), metaQTLs (1321), epistatic QTLs (202) in Triticum aestivum and seven other related wheat species [154]. The QTLs identified in different crop species for different abiotic stress parameters are provided in Table 2. The breakthroughs in sequencing technologies also enable a quick and cost-effective technique for obtaining a large number of varied ESTs that could aid in the computational identification of molecular markers such as SNPs and SSRs in food crops. These markers can enable the (MAS) selection of important agronomic traits for high yield and abiotic stress tolerance. It requires the validation of QTLs, which can be overcome by functional markers. This massive number of markers is a valuable resource for facilitating breeding food crop lines with abiotic stress tolerance, including drought, extreme temperature, and salinity stresses.

### 3.3. Genome-Wide Association Studies (GWAS)

GWAS is a powerful tool because it can determine natural variations in all the recombination events that occur in the evolutionary processes of a wide range of organisms [170,171]. The main objective of the GWAS is to identify SNPs [172]. WGAS exploits the databases that contain reference plant genome sequences and genetic maps to analyse whole samples to find genetic variations based on complex traits such as growth rate, flowering time, and yield. They are the major focus of crops to improve the quality and understand the adaptation of plants. This study revealed DNA samples of tolerant and susceptible plant varieties on a chip and they were scanned through an automated scanning machine. It can search the genetic variations for a selected marker such as SNPs if the variations associated with traits are found. One of the most important factors in GWAS is data quality. PLINK, a freely available tool set, can be used. It contains two data sets: one consists of individuals and their genotypes, and the other contains information on the genetic markers [172]. PLINK provides Principal Component Analysis (PCA) for population stratification and Bonferroni correction to control the false discovery rate. SNPs could be used in a PRS (Polygenic Risk Prediction Analyses) analysis. Genotypes with a *p value* > 5% missing rate and a *p value* < 1% allele frequency create problems for further analysis and thus are eliminated from the study [91]. GWASs were completed successfully on food crops such as rice, maize, sorghum, and foxtail millet. Huang et al. [173] identified approximately 3.6 million SNPs in 517 rice land races by GWAS. GWAS of 950 accessions of cultivar led to the identification of loci associated with flowering time and grain yield. They were genotyped into the rice land races by the sequencing-by-synthesis method. GWAS of *S. bicolor* was performed on 971 accessions, yielding approximately 265,000 SNPs. Loci were associated with height and maturity [174]. Using GWAS, Jia et al. [175] discovered 2.58 million SNPs in 916 foxtail millet (*Setaria italica*) varieties. This study grouped 916 foxtail millet varieties into five categories, and 512 loci are related to 47 agronomic traits. GWAS will allow comparative studies of adaptive genetic variation among species that have potentially evolved in parallel under selective stress. A better understanding of the adaptive responses of plants under stressed conditions might be obtained by comparing the genetic structures of adaptive traits among species.

## 4. Transcriptome and Gene Expression Analysis of Abiotic Stress Responses in Cereals

**Figure 4:**
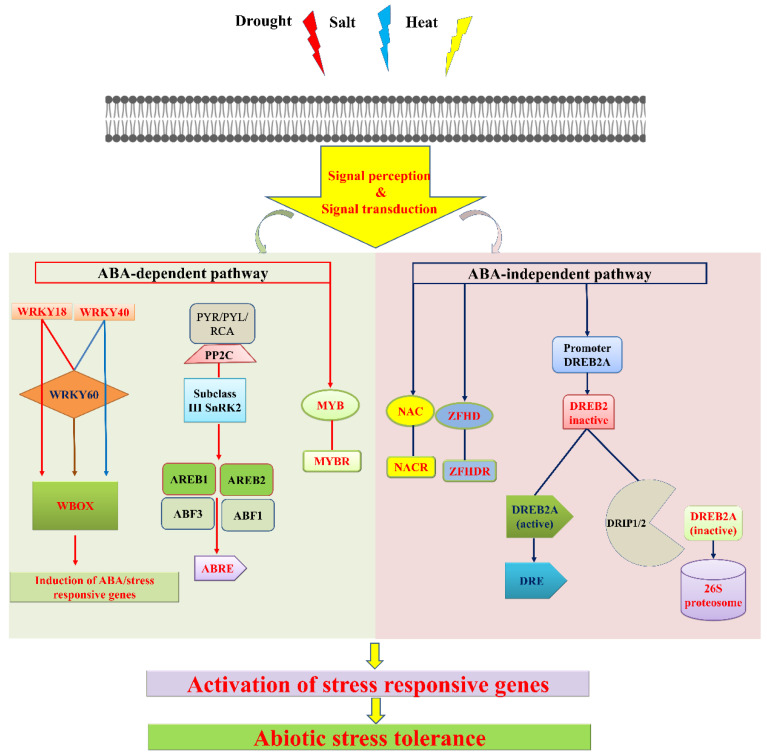
Schematic representation describes the ABA-dependent and independent signaling pathways involved in acquiring drought, salt and heat stress responses, and tolerance. ABA—abscisic acid; ABRE-ABA-responsive element; AREB-ABRE-binding protein; ABFs-ABRE-binding factors; DREB2-DRE-/CRT-binding protein 2; DRE-dehydration-responsive element; SnRK2-SNF1-related kinase 2; DRIP1-DREB2A-interacting protein 1; MYB-MYB proto-oncogene transcription factor; NAC—no apical meristem transcription factor; PP2C—protein phosphatases 2C; PYR—pyrabactin resistance 1; PYL—pyr1-like; RCA—regulatory components of ABA receptor; ZFHDR—zinc finger homeodomain transcription factor.

Transcriptomics is the analysis of gene expression profiles able to reveal genes that are responsible for abiotic stress. Understanding such responses could be crucial for effectively managing abiotic stress. Transcriptome profiling provides an opportunity to investigate the functional characterization of individual genes in stress tolerance mechanisms. In recent years, more next-generation sequencing (NGS) and RNA sequences for sRNAs have been generated for improved plant genomic resources [176,177]. The high-throughput transcriptome sequencing dataset provides an efficient strategy for gene discovery, molecular marker development, and marker-assisted breeding [178]. Typical plant responses to abiotic stress involve a network of molecules. ABA (abscisic acid) is a major phytomolecule that plays an important role in responding to a variety of stresses such as drought, heat stress, high salinity, heavy metal stress, low temperature etc., They are also involved in various developmental processes, including seed germination, seed dormancy, and closure of stomata. ABA-dependent pathways appear to recruit antioxidant and osmoprotectant mechanisms, ABA-independent pathways generally involve protective proteins [179,180]. ABA-dependent and ABA-independent pathways involved in the acquisition of drought, salt, and heat stress responses and tolerance are shown in Figure 4. Details on comparative transcriptome analysis studies reported for cereals are given in Table 3. Such studies are helpful for the identification of genes, hormones, and processes related to drought, heat, and temperature stress. 

### 4.1. Drought Stress

The comparative analysis of plant responses to survival under water-deficit conditions and several transcription factors that regulate stress-inducible gene expression were identified in rice with Arabidopsis by external ABA treatment [194]. However, the expression of stress-related genes depends on the interaction between transcription factors and their respective promoter cis-elements. Stockinger et al. [195] found that in Arabidopsis, transcription factor DREBs specifically bind to ABRE (ABA-responsive element) and stimulate the expression of ABA-responsive genes. In rice, five DREB homologs were identified: *OsDREB1A*, *OsDREB1B*, *OsDREB1C*, *OsDREB1D*, and *OsDREB2A* [196,197]. They are also involved in transcriptional regulation of the cold and salt stress-response mechanisms [198]. Lenka et al. [199] conducted an experiment involving drought-tolerant and drought-sensitive cultivars to identify novel gene regulatory mechanisms. They also discovered upregulated genes associated with the linolenic acid metabolic pathway in tolerant genotypes. Similarly, RNA-Seq analysis has been carried out between wet and dry soil sowing rice seedling roots and found an enzyme called RING box E3 ligases, which involves the ubiquitin–proteasome pathway [200]. RNA-Seq analysis has been completed by Ma et al. [201] to investigate the effect of drought on the wheat genome during the reproductive stages of wheat. This study reveals the 309 differentially expressed genes (DEGs) that respond to various developmental stages of wheat. At early developmental stages, the proteins of these DEGs were mainly located in the nucleus, peroxisome, mitochondria, plasma membrane, and chloroplast, indicating that these organelles play critical roles in drought tolerance in wheat. 

Li et al. [202] reveal the transcriptome profile of B73 (maize) seedling leaves exposed to drought, salinity, heat, and cold stress using the Illumina sequencing system. This study suggests a common response initiated in maize under different abiotic stresses (drought, salinity, heat, and cold). In addition, TFs genes involved in stress response, five ERFs, two NACs, one ARF, one MYB, and one HD-ZIP family were up-regulated, and one b-ZIP and one MYB family were downregulated. Furthermore, transcriptome analysis was completed at the flowering time of *Zea mays* L. under drought stress using RNA-seq and bioinformatics tools. They identified 619 genes and 126 transcripts, and their expression was altered due to drought stress. Comparative transcriptome analysis also found the differentially expressed genes in maize between the tolerant and sensitive varieties of seven-day drought-treated plants [203]. The transcriptional factors such as MYB, NAC, WRKY, and PLATZ were modulated, which involved carbohydrate synthesis, cell-wall remodeling, amino acid biosynthesis, and protein ubiquitination processes. This discovery of drought-responsive genes lays the groundwork for further targeted cloning and downstream analysis of the specific individual genes. In *Sorghum bicolor*, combined heat and drought stress responses were analyzed using a microarray. The results exhibit the specificity and cross-talk between the sorghum response under individual and combined stress [191]. Chinese barley was found to have differentially expressed genes compared to the control and different relative soil moisture content (RSMC) plants [204]. The up-regulated genes of low RSMC plants were mainly involved in ABA-dependent and independent pathways, which include NCED, PYR, ABF, MYB/MYC, SnRK2, LEA, ERF family, and DHN. Shi et al. [205] revealed the photosynthetic metabolic pathway of the drought-resistant F_1_ hybrid (M79) and its parental lines (E1 × H1). RNA-sequence analysis of these foxtail millet lines showed the photosynthesis-related genes were highly expressed in the F_1_ hybrid compared to its parental lines.

### 4.2. Heat Stress

Heat Shock Protein (HSPs) expression is a common factor in plants when they undergo heat stress. HSPs are regulated by heat stress transcription factors (HSFs). HSEs in HSP gene promoters are highly conserved and consist of inverted repeats of the palindromic motifs of nGAAn [206,207]. Ikeda et al. [208] found that heat stress increases the expression of HSFs in *Arabidopsis*. Overexpression of the HSF gene in *Arabidopsis* plants improved heat tolerance [209]. The expression of the *OsHSFA2a* gene is highly stimulated by heat stress, particularly in root and shoot tissues of rice plants during seed and panicle development [210]. In wheat, overexpression of *TaHsfA6f* in transgenic wheat plants showed improved heat tolerance [211]. 

An Affymetrix 22K Barley1 GeneChip microarray was used to investigate the heat stress response of barley caryopses. The microarray results provided information about the differentially regulated genes such as *Raffinose synthase 1, UDPd-glucose 4-epimerase 1, UDP-d-glucose 4-epimerase 3, trehalose-6-phosphate synthase, trehalose-6-phosphate phosphatase, invertase inhibitor, heat shock transcription factor A2d, hexokinase 2,* and *SNF1-related protein kinases* [192]. Lin et al. [189] identified all the HSF genes in maize such *as ZmHsf-01, ZmHsf-03, ZmHsf-04, ZmHsf-06, ZmHsf-10, ZmHsf-11, ZmHsf-14, ZmHsf-15, ZmHsf-19, ZmHsf-20, ZmHsf-21, ZmHsf-22, ZmHsf-23, ZmHsf-24, and ZmHsf-25* under heat stress using qPCR. This knowledge could be beneficial in the improvement of strategies to produce crop plants with a tolerance to heat stress.

Jung et al. [183], using the NSF45K array in rice, identified 244 genes (early heat stress) and 238 genes (prolonged heat stress), including (histone deacetylase) HDAC (Os06g38470) with 4 HSFs (Os01g54550, Os03g12370, Os04g48030, and Os08g43334) in early heat stress response and HDAC (Os02g12350) with 3 HSFs (Os03g53340, Os09g35790, and Os10g28340) in prolonged heat stress response. A comparative heat stress response transcriptome analysis was carried out in rice primarily based on Illumina RNA-Seq. The study revealed the expression of differentially regulated genes at 0 h, 12 h, and 24 h. Among these, 1599 genes were up-regulated, and 1754 genes were downregulated in the HT (heat tolerant) line at 24 h, but in the HS (heat susceptible) line, inconsistent results were shown and 1438 genes were up-regulated, and 1237 genes were downregulated [212]. Transcriptome studies have provided a fast and simple method to study the transcript levels of heat stress tolerance mechanisms and crop genetic improvement in cereals.

### 4.3. Salinity Stress

Transcriptomic approaches provide new insights for a better understanding of the molecular mechanisms involved in salt stress in food crops. The novel molecular stress response and plenty of unreported salt stress-responsive genes are provided by the meta-transcriptome analysis of rice (TNG67) genotypes under salt stress. It can shift the fermentation pathway to produce energy to enhance the Calvin cycle to repair a damaged photosystem under salt stress [213]. Comparative transcriptome analysis also found differentially expressed genes in rice between the tolerant and sensitive genotypes. The results showed the 286 DEGs in a tolerant genotype at both 48 h and 72 h, and in sensitive genotypes, DEGs 185, 156, and 909 at both the 48 h and 72 h time points, respectively. The differentially expressed genes involved in a salt-tolerant response included peroxidase activity, chromatin binding, ATP binding, heme binding, transferase activity, transferring hexosyl groups, magnesium ion binding, protein serine/threonine phosphatase activity, and cysteine-type endopeptidase activity. 

Comparative transcriptome analysis also found the differentially expressed genes in bread wheat between the salinity tolerant and sensitive cultivars after 150 mM salinity treatment. The results show that 5128 genes were differentially expressed between salt-treated and control plants. Among them, 1995 genes were upregulated, and 3133 genes were downregulated. Furthermore, 109 and 210 genes were found to be specifically expressed in salt-treated and control plants, respectively [214].

Osthoff et al. [161] revealed the effects of water deficit, salinity stress, and combined stress. In this study, transcriptome sequencing was performed at 6 h and 24 h after stress treatment. The gene expression responses observed at 6 h of stress also remain at 24 h of stress. However, after 24 h of stress, hundreds of genes were additionally regulated compared to 6 h of stress treatment. After 6 h salt treatment, 953 genes were differentially expressed; 1802 genes were expressed after 24 h salt treatment. All three stresses, transcription factors bHLH (basic Helix-Loop-Helix), ERF (Ethylene Response Factors), and HSF (Heat Shock Factors), have been enriched at each time point (6 h and 24 h). In addition, the bZIP was enriched after both water deficits and combined stress at both time points. Salt stress, bZIP, G2-like, and HD-ZIP TFs were enriched after 24 h of treatment.

Whole-genome transcriptomics (WGT) analysis of “Chilbo” rice cultivar under 250 mM NaCl treatment was carried out. After 12 days of treatment, 962 upregulated genes were identified, most of the genes belong to the MYB family and ZF family. These gene families are majorly involved in the regulation of sugar metabolism and amino-acid synthesis [215]. Comparative RNA-seq analysis of two rice cultivars, Dongdao-4 and Jigeng-88, were carried out by Li et al. [216]. They revealed 3523 and 4066 differentially expressed genes responding to several gene families, which includes jasmonic acid, organic acid metabolism, phenylpropanoid, gibberellic acid synthesis, and iron homeostasis.

Ma et al. [217] found upregulation of TaCYP450 in wheat plants under salt stress. Similarly, LEA, dehydrin and potassium transporter genes in roots, and sodium/cation exchanger and aquaporin genes, ETHYLENE RESPONSE FACTORs (ERFs) (TaERF1, 2, 3, 4, and 6) shoots were upregulated in wheat plants. Furthermore, genes encoding expansin, xyloglucan endotransglucosylase/hydrolase, dehydrins, peroxidases, and TFs WRKY, MYB, NAC, bHLH, AP2/ERF also found to be upregulated under salt stress [218,219].

Comparative transcriptome analyses of maize seedling root responses to salt stress revealed the upregulation of Aux/IAA, SAUR, CBL-interacting kinase, ABA signal pathway, WRKY, bZIP, and MYB in tolerant cultivar. The ABA biosynthesis gene, GRMZM2G127139 ABA1/LOS6/ZEP, was consistently repressed and one ABA-responsive gene, GRMZM2G162659 (EM1), was upregulated [220,221].

## 5. Proteomics Approaches to Identify the Functional and Structural Characteristics of the Abiotic Stress-Responsive Proteins in Cereal Crops

Proteomics has been successfully used to analyze protein profiles and their functions, and it is considered as one of the next-generation research tools in life science, along with genomics and transcriptomic [222,223]. Proteins are the important biomolecules responsible for all cellular functions. Plant proteomes are complex and dynamic, and dealing with them is difficult. It is critical to select appropriate proteomic techniques for protein identification and modification that may contribute to crop development. Recent advancements in quantitative proteomics research utilizing high-resolution and mass accuracy instruments have contributed to the identification of proteins and their expression profiles, post-translational modifications (PTMs), and protein–protein interactions under stressed and normal conditions [4,224,225]. Plant proteomic analysis is achieved through systematic high-throughput methodologies, including 2D-PAGE (2-Dimensional-Poly Acrylamide Gel-Electrophoresis), MALDI-TOF/MS/MS, and LC-MS/MS. Elucidation of protein functions and functional protein networks are evaluated in plant metabolic and signaling pathways using protein mapping, characterization of PTMs, protein-protein interaction, and systems biology strategies [4,226]. Additionally, proteomic databases were developed for rice, maize, and wheat, including PhosphoRice (http://bioinformatics.fafu.edu.cn/PhosphoRice (accessed on 21 January 2022)) [227], Oryza PG-DB: (http://oryzapg.iab.keio.ac.jp/ (accessed on 23 January 2022)) [228], PRIN: rice proteome database (http://bis.zju.edu.cn/prin/ (accessed on 5 February 2022)) [229], PPDP: Arabidopsis and maize proteome database (http://ppdb.tc.cornell.edu (accessed on 10 March 2022)) [230], and wheat proteome (http://www.wheatproteome.org/ (accessed on 20 April 2022)) [231]. Recently, the maize genomics and genomic database (https://www.maizegdb.org/ (accessed on 4 October 2022)) and the International rice informatics consortium (http://iric.irri.org/resources/rice-databases (accessed on 4 October 2022)) have been developed. Furthermore, databases are already available for plant metabolic (https://plantcyc.org (accessed on 5 October 2022)) and proteomic (http://ppdb.tc.cornell.edu/ (accessed on 5 October 2022)). The plant proteomic database allows us to collect information about protein function, properties, and subcellular localization. 

These bioinformatics resources help to categorize the identified proteins into functional categories and their dynamic interaction networks with other plants. Proteomics approaches help to increase the nutritional value and yield of crops and also help to understand the crop adaptation mechanisms of crops under abiotic stresses. 

### 5.1. Drought Stress

Proteomic approaches provide new investigation methods for a better understanding of rice’s molecular mechanisms involved in drought stress. Mirzaei et al. [232] identified 900 proteins in rice roots during drought stress. Among them, 38% were changed in abundance compared to the non-treated. The proteins involved in pathogenesis-related chitinases and redox proteins were increased, while tubulins and transport-related proteins were decreased. A comparative proteome analysis of drought tolerant (IRAT109) and sensitive (Zhenshan97B) varieties was carried out. The tolerant variety showed increased protein abundance (14 proteins in increased abundance; 6 proteins in decreased abundance) compared to the susceptible variety (2 proteins in increased abundance; 15 proteins in decreased abundance). IRAT109 was protected by proteins involved in superoxide dismutase and dehydroascorbate reductase enzyme activity [233]. 

To investigate the effect of PEG (6000) induced drought stress on three rice cultivars, drought tolerant (NSG19), susceptible (IR20), and intermediate tolerance (KDML105), proteomes were compared using LC-MS/MS. In this study, 623 proteins were identified, and only 53 proteins showed significant abundance between the three cultivars. The drought-tolerant variety’s highly abundant proteins are involved in cell and DNA repair and stomatal closure [234].

Based on grain yield ability during drought stress, iTRAQ-based quantitative proteome analysis was performed in three different wheat cultivars: RAC875 (drought tolerant), Excalibur (drought-tolerant), and Kukri (drought-intolerant), subjected to cyclic drought conditions [235]. These cultivars at different time points of drought stress showed distinct physiological responses, with similarly drought tolerant varieties (RAC875, Excalibur) differing in their protein responses. RAC875 proteins showed significantly abundant proteins compared to Excalibur, which lacked significant proteins. At the same time, all three cultivars had a protein involved in oxidative stress metabolism and ROS scavenging capacity to increase SOD and CAT enzyme activities. However, both drought tolerant cultivars are involved in ROS avoidance through decreased proteins responsible for photosynthesis and the Calvin cycle. This study suggests that each wheat species has a unique mechanism to tolerate drought stress.

Alvarez et al. [236] compared the root proteome of contrasting wheat genotypes [Nessar-tolerant; Opata M85-sensitive] in the presence and absence of (abscisic acid) ABA using a similar iTRAQ-based quantitative proteomics approach. The study reveals the proteome expression levels between Nesser and Opata roots responses to ABA. More ABA-responsive proteins were found in Nesser compared to the Opata root. In PEG-induced drought stress and recovery, the upregulated HSPs chaperone family members were found in drought-tolerant wheat cultivar Hanxuan 10 compared to drought-sensitive cultivar Chinese Spring plants. The phosphorylation level of these proteins was also increased [237].

Deng et al. [238] studied the proteome of wheat flag leaves and developing grains in response to drought stress and identified 87 DAPs in flag leaves and 132 differentially accumulated protein spots (DAPs) in developing grains. DAPs related to oxidative stress were significantly upregulated in developing grains and downregulated in flag leaves. While photosynthesis and starch biosynthesis DAPs were upregulated in flag leaves, they were downregulated in developing grains. Drought significantly reduced photosynthesis in leaves and carbon metabolism in grains, thus resulting in a significant decrease in starch biosynthesis and grain yield.

To investigate the effect of drought on the maize leaf proteome, iTRAQ and 2-DGE analyses were performed by Benešová et al. [239]. This study showed both genotypes upregulation of protective and stress-related proteins (chaperones and dehydrins). It revealed that 106 out of 220 differentially expressed and identified proteins were up-regulated in the tolerant genotype and downregulated in the sensitive genotype. The drought tolerant genotype CE704 showed upregulation in protective and detoxification proteins such as ascorbate peroxidase, superoxide dismutase, glutathione reductase, and catalase. Furthermore, EF-TuM, a mitochondrial translation elongation factor, and eIF3, a translation initiation factor, were also upregulated. 

The proteomic profile of maize leaves was identified and quantified using a multiplex iTRAQ-based quantitative method. Expression levels of 135, 65, and 201 proteins were significantly changed under the heat, drought, and combined stress conditions, respectively. Among these, 61, 28, and 16 responded exclusively to drought, heat, and combined stress, respectively. HSPs showed abundant expression under heat stress and combined stress, suggesting that HSPs play a crucial role in maize tolerance to heat stress and combined stress [240].

Comparative proteome analysis also found differentially accumulated protein spots in maize between the tolerant [YE8112] and sensitive [MO17] genotypes to drought stress [203]. They identified a total of 721 differentially abundant proteins (DAPs). They identified a total of 721 differentially abundant proteins (DAPs). Among them, 13 DAPs specific to YE8112, 84 DAPs unique to MO17, and 107 specific DAPs shared between drought-sensitive and drought-tolerant lines after drought treatment. The upregulated proteins of the tolerant genotype (YE8112) activated photosynthesis (PSII), lipid-metabolism, and stimulated chaperons such as ASR1 protein. 

Jedmowski et al. [241] compared the drought tolerance of two barley cultivars. They found that the proteins were upregulated in the drought-tolerant cultivar. These include lipooxygenase, NADP-dependent malic enzyme, sucrose synthase, and betaine aldehyde dehydrogenase. Other proteins elevated in both the cultivars were methionine synthase, ATP synthase alpha subunit, HSP 90, aconitase, ATP-dependent CLp protease, alanine glyoxylate aminotransferase, and protein disulfide isomerase. These included elongation factor EF2, metalloprotease, HSP 70, and a stress-sensitive protein in the tolerant cultivar. In the sensitive cultivar, leucine aminopeptidase, lipoxygenase, sucrose synthase, betaine aldehyde dehydrogenase, and NADP-malic enzyme were increased.

Similarly, Wang et al. [242] performed a comparative proteomic study on drought stress in Tibetan wild barley genotypes (drought tolerant XZ5 and drought-sensitive XZ54) and cv. ZAU 3. The 38 drought-tolerance-associated proteins identified in this study were involved in the functional categories of the stress response, photosynthesis, metabolic process, energy, and amino acid biosynthesis. Out of 38 proteins, 20 were up-regulated in XZ5 and simultaneously downregulated in XZ54, highlighting the significance of “drought-tolerance-associated specific proteins” in drought tolerance [242].

Comparative proteomic analysis of two *Sorghum* cultivars (Accession No. 11434, drought tolerant, and Accession No. 11431, drought sensitive) led to the identification of differentially expressed proteins under drought stress [241]. Proteins involved in the energy balance, metabolism, and chaperons were altered in the tolerant and sensitive cultivars. Methionine synthase was upregulated in both the genotypes (#11434 and #11431) under drought stress following recovery. However, pyruvate phosphate dikinase (PPDK) was upregulated only in the tolerant cultivar. Ref. [243] investigated the effect of drought stress on foxtail millet. They identified 321 proteins that were differentially expressed between drought-treated and control plants. The results showed that, out of 321 proteins, 252 proteins were upregulated, and 69 were downregulated. The identified proteins involved in the stress responses include photosynthesis, carbon metabolism, ROS scavenging, fatty acid, and amino acid metabolism, polyamine biosynthesis, hormone metabolism, and cell wall modifications.

### 5.2. Heat Stress

The heat stress response in plants is a very complex process involving the upregulation and downregulation of numerous proteins involved in plant protection, protein biosynthesis, protein degradation, energy, carbohydrate metabolism, and redox homeostasis. Experiments were carried out to understand the mechanisms behind heat stress, and a significant change in the abundance of protein was observed in rice after 1, 4, and 5 days of heat stress. The protein variation between the tolerant and sensitive plants was compared using MALDI-TOF/TOF MS [244]. This study reveals the 27 differentially expressed proteins. Out of 27 proteins, 25 were homologous to known functional proteins. In this study, GDI (GDP dissociation inhibitor), ADPase (ADP-glucose pyrophosphorylase), and UDPase (UDP-glucose pyrophosphorylase) were up-regulated in heat-tolerant and heat-sensitive rice after 3 days of high temperature conditions but downregulated in sensitive rice after 5 days of heat stress. Similarly, heat shock proteins, oxidation, and biosynthesis proteins were up-regulated in tolerant cultivars and downregulated in sensitive cultivars after five days of stress [244]). 

Mu et al. [245] studied the effects at the anthesis stage of two contrasting rice cultivars (N22-tolerant; Mianhui101-sensitive) after exposure to heat stress. The study reveals differences between the two cultivars in the differentially expressed proteins from both tolerant and sensitive cultivars. Specifically, many ribosomal proteins were downregulated in the sensitive cultivar (Mianhui101), but contrastingly, sHSPs, -expansins, and lipid transfer proteins were increased in the sensitive cultivar. These proteins were also upregulated in the tolerant to N22 cultivar, which may be involved in the heat tolerance. 

In wheat, 256 differentially expressed proteins were identified under heat stress. HSP90, PDI (protein disulfide isomerase), Myb-related proteins, the 40S, and 60S ribosomal proteins, and DnaJ protein were up-regulated. ADPG-PPase (adenosine diphosphoglucose pyrophosphorylase), PFP (pyrophosphate-fructose-6-phosphate1-phosphotransferase), sucrose synthase, and SBEIIb (starch branching enzyme IIb) were downregulated [246]. Heat stress-associated active proteins (SAAPs) were quantified in two different wheat cultivars (HD2985-heat tolerant; HD2329-heat sensitive) using iTRAQ [247]. They identified differentially expressed 4272 SAAPs, RuBisCo, Rca, OEEP, HSP17, SODFe/Zn-SOD, gamma gliadin, and peroxidase. These were upregulated in the tolerant line (HD2985) and downregulated in the sensitive line (HD2329). Proteome analysis performed in maize plants determined the changes in protein types and their expression levels under heat stress [248]. They found 59 and 104 protein spots by 2D analysis after 2 and 4 hr of high-temperature treatment, respectively. Furthermore, these protein spots were quantified by LC-MS/MS and identified as ATPase beta subunit, HSP26, HSP16.9, and unknown HSP/Chaperonin.

### 5.3. Salinity Stress

Xu et al. [249] investigated rice’s responses to salt (150 mM NaCl) stress using a proteomic approach. They found 56 proteins significantly changed, and 16 of them enhanced the photosynthesis, antioxidant, and oxidative phosphorylation pathways through the upregulation of the peroxiredoxin Q and photosystem I subunit D. Salt exposure triggered a strong decrease in the abundances of thioredoxin x, thioredoxin peroxidase, glutathione S-transferase F3, PSI subunit H, light-harvesting antenna complex I subunits, vacuolar ATP synthase subunit H, chloroplast chaperonin, and ATP synthase delta chain. Zhao et al. [240] revealed the phosphoproteomic differences between a salt-tolerant (Zheng58) and a salt-sensitive (Chang7-2) maize cultivar during short-term salt stress. In response to salt stress, 209 and 243 differentially regulated phosphoproteins (DRPPs) were identified in root and shoot, respectively. These DRPPs include 6-phosphogluconate dehydrogenase 2, pyruvate dehydrogenase, phosphoenolpyruvate carboxykinase, glutamate decarboxylase, glutamate synthase, l-gluconolactone oxidase-like, potassium channel AKT1, high-affinity potassium transporter, sodium/hydrogen exchanger, and calcium/proton exchanger CAX1-like protein in roots. In contrast, phosphoenolpyruvate carboxylase 1, phosphoenolpyruvate carboxykinase, sodium/hydrogen exchanger, plasma membrane intrinsic protein 2, glutathione transferases, and abscisic acid-insensitive 5-like protein were significantly upregulated in shoots [240].

After salt treatment with 200 mM NaCl in two contrasting rice cultivars, Pokkali (tolerant) and IR64 (sensitive), the proteome analysis was performed using the iTRAQ approach [250]. A higher abundance of proteins was found in the tolerant cultivar Pokkali in comparison to the sensitive cultivar IR64. The identified proteins are involved in stress tolerance (ascorbate peroxidase, superoxide dismutase, peptidyl-prolyl cis-trans isomerases and glyoxalase II) and photosynthesis (oxygen evolving enhancer proteins OEE1 and OEE3, PsbP) [250].

Comparative proteome analysis of two inbred maize lines (salt-tolerant 8723 and salt-sensitive P138) reveals a large number of salt-responsive DEPs involved in phenylpropanoid biosynthesis, starch, and sucrose metabolism, and the mitogen-activated protein kinase (MAPK) signaling pathway in 8723 maize lines, but only the nitrogen metabolism pathway DEPs present in P138. This study exhibits a tolerant capacity of 8723 compared to P138. This is because 8723 can preserve the osmotic regulation ability, synergistic effects of antioxidant enzymes, a stronger water retention capacity, energy supply capacity, signal transduction, ammonia detoxification ability, lipid metabolism, and nucleic acid synthesis [251]. Rasoulnia et al. [252] investigated salinity-responsive total proteins in different barley genotypes by comparing leaf proteins from the salt-tolerant (Afzal) cultivar to the salt-sensitive (L-527) barley genotype exposed to short-term salt stress. A total of 117 salt-responsive proteins were detected in the two genotypes. The tolerant cultivar showed more protein abundance than the sensitive cultivar. At the same time, the salt-sensitive genotype showed a higher number of proteins with decreased abundance. Similarly, Fatehi et al. [253] also studied salt-responsive proteins in the two genotypes under long-term salt stress and identified 44 salt-responsive proteins. Up-regulated proteins were involved in reactive oxygen species scavenging, signal transduction, protein processing, and cell wall formation, which may increase plant adaptation to salt stress. The antioxidant proteins, thioredoxin, methionine sulfoxide reductase, and dehydroascorbate reductase were upregulated in both genotypes. Under 100 mM NaCl stress, proteins involved in signal transduction (annexin, translationally-controlled tumor protein homolog, lipoxygenases), detoxification (osmotin, vacuolar ATP-ase), protein folding (protein disulfide isomerase), and cell wall metabolism (UDP-glucuronic acid decarboxylase, -d-glucan exohydrolase, UDP-glucose pyrophosphorylase) were upregulated [254].

The exogenous application of melatonin increased salt tolerance. A transcriptomics study indicated that the melatonin-mediated pathway contributed to salt tolerance, specifically AP2/EREBP-HB-WRKY transcriptional cascade and phytohormone (auxin and ABA). Protein analysis of ‘F63’ (tolerant) and ‘F35’ (sensitive) maize genotypes using the iTRAQ approach identified 28 salt-responsive proteins, of which 22 were expressed explicitly in ‘F63’ [255]. Chen et al. [251] identified 1056 DEPs, of which 626 and 473 were specific to tolerant and sensitive maize inbred lines, respectively. DEPs expressed in the tolerant lines under salt stress were associated with phenylpropanoid biosynthesis, starch and sucrose metabolism, the MAPK signaling pathway, ribosomal proteins (RPs), nucleoside diphosphate kinases (CDPKs), transaldolases (TALs), beta-glucosidases (BGLUs), phosphoenolpyruvate carboxylases (PEPCs) DAPs associated with the Calvin cycle, amino-acid metabolism, carbon and nitrogen metabolism, transcription and translation and antioxidation.

Comparative proteome analysis of two maize cultivar, PH6WC (tolerant) PH4CV (sensitive), was carried out by metabolomic assay. Nucleic acid metabolism was significantly higher in the salt tolerant genotype. Furthermore, some compounds were increased under salinity such as cis-9-palmitoleic acid, L-pyroglutamic acid, galactinol, deoxyadenosine, and adenine [256]. 

## 6. Future Perspectives

Important cereal plants such as rice, wheat, maize, barley, sorghum, and millets (foxtail millet, pearl millet, finger millet, etc.) are major food crops, contributing to 92% of total crop production across the agricultural field and in almost all countries. Abiotic stress, such as drought, high temperatures, and salinity, is a major problem for the production of food crops and can result in reduced yields and lowered quality. Therefore, the development of biotic and abiotic stress-tolerant plants (including cereals) through molecular breeding helps to enhance plant growth and yield under biotic and abiotic stresses. Compared to the other techniques of plant molecular biology, molecular breeding techniques (especially QTL identification and validation) are favorable and an ancestor for cereals growth and development. This is because this was reported to be improve various agro-morphological, nutrients contents and yield traits in cereals under biotic and abiotic stresses. Progress has been made in omics technologies to understand the abiotic stress response and has considerably improved. The comprehensive structure of traditional omics studies opens up an entirely new avenue for understanding the molecular mechanisms of plant responses to abiotic stress. Under stress conditions, plants modulate themselves to adopt the existing stresses by controlling gene regulation, proteins, and metabolites. It is essential to elucidate the functions of newly identified stress-responsive genes to understand the abiotic stress responses of plants. Various omics approaches such as genomics, transcriptomics, metabolomics, ionomics, and phenomics have been devised to allow the understanding of genetic makeup in depth, their signalling cascade, and their adaptability under stress conditions.

Future research programs should aim to adapt to omics study. In most of the cereal crops, genomics and transcriptomics have progressed as expected but the other major omic branches such as proteomics, metabolomics, phenomics and ionomics are still lagging. These omics branches are equally crucial to understanding the biological system. The field of omics is expanding, and numerous new omics topics are expected to emerge soon. It will help provide better quality food for human consumption and improve human health. 

The integration of multiple omics studies has revealed new areas of interactions and regulation. To obtain a comprehensive understanding of plant responses to abiotic stress, more extensive mapping of these responses at the organ, tissue and cellular level are needed. Such network analyses need to be extended to the proteomics and enzyme activity. Models need to be constructed and linked to phenotypic traits. The linkage of key regulatory hubs to phenotypic traits will allow for more rapid progress in the genetic manipulation and production of cereal crops. Functional genomics approaches have identified the role of various abiotic stress-responsive genes in cereals. Nowadays, several plant researchers predominantly use the already well-established tool clustered, regularly inter-spaced, short palindromic repeats (CRISPR)/CRISPR associated protein (Cas) (CRISPR/Cas). Therefore, developing plants by CRISPR/Cas variants (base editor, primer editor, CRISPR activator, and CRISPR repressor) helps improve the cereals’ growth and yield under abiotic stresses. 

## Figures and Tables

**Table 2:** QTL identified in different cereal crops for abiotic stress-related traits.

Crop (Common Name)	QTL Trait Identified	Stress	Trait Improved	References
Rice	Seed weight and grain yield, osmotic potential, water potential, cell membrane stability	Drought	Yield, grain, plant biomass, root length	[25,155,156,157]
Maize	Leaf growth, ionic balance, osmotic adjustment	Drought	Yield and grain	[158,159,160]
Sorghum	Water-related attributes, ionic balance, osmotic adjustment	Drought	Delayed leaf senescence, yield and grain	[161]
Pearl millet	Osmotic potential, water-related attributes, cell membrane stability	Drought	Yield and grain	[162]
Wheat	Osmotic potential, seed weight and grain yield, salt tolerance	Drought, cold, salinity, metal toxicity, nutrient deficiency, heat	Plant biomass, early flower and maturity, yield	[163,164,165,166]
Barley	Water potential, osmotic potential, freezing tolerance	Drought, salinity, water logging, metal toxicity	Grain	[167,168,169]

**Table 3:** Transcriptomics approaches to understanding the heat-stress induced responses in important cereals.

Plant Species	Approach Used	Differentially Regulated Genes	Reference
Rice	Microarray	HsfA2a, HsfA2d, HsfA2f, HsfA3, HsfB2a, Hsfb, Hsfc, DREB, ERF, and members of HSP70, HSP90, and HSP100	[103]
	Microarray	Microarray Hsfs, sHSPs, members of HSP70, HSP90, and HSP100 gene families	[181]
	RT-PCR	OsHsfA4b, OsHsfA5, OsHsfA7, OsHsfA4d, OsHsf A2a, OsHsfA2c, and OsHsfA2d	[182]
	Microarray	Hsfs, bZIP TFs, HSP10s, and HSP20s	[183]
	RNA-seq, qRT–PCR	sHSP genes, HSP101 or heat shock factor (HSF) genes, TFs- WRKY, MYB, AP2/ERF	[184]
	RNA-seq, qRT–PCR	HSP 20 and HSP70 family), heat shock protein binding protein 1 (HSPBP1, HSP70-interacting protein), DREB, RAB, and late embryogenesis abundant (LEA) proteins	[184]
Wheat	Microarray	Hsfs, HSPs, DREB2B and DREB6A, ERETC, and member of MBF1	[185]
	Microarray	Wheat microarray b-ZIP transcription factors andTaCAM3-1(zinc finger with calmodulin)	[186]
	Microarray	HSPs, transporters, protein modifiers, and signaling molecules	[187]
	qRT-PCR	Chloroplast-localized small heat shock proteins (sHSP) encoded by the Hsp26 gene	[188]
Maize	qRT-PCR, RNA Sequence	ZmHsf-01, ZmHsf-03, ZmHsf-04, ZmHsf-06, ZmHsf-10, ZmHsf-11, ZmHsf-14, ZmHsf-15, ZmHsf-19, ZmHsf-20, ZmHsf-21, ZmHsf-22, ZmHsf-23, ZmHsf-24, and ZmHsf-25	[189]
		HSP 26, HSP 70, sHSP Cytokinin, signal transduction and DNA synthesis/chromatin structure	[190]
		Photosynthesis, oxidation-reduction process, peptidase inhibitor activity, peptidase regulator activity and inositol tetrakisphosphate kinase activity	[190]
Sorghum	Microarray	TFs-MYB78 and ATAF1, chaperones.	[191]
Barley	Microarray, RNA Sequence	Raffinose synthase 1, UDPd-glucose 4-epimerase 1, UDP-d-glucose 4-epimerase 3, rehalose-6-phosphate synthase, trehalose-6-phosphate phosphatase, invertase inhibitor, heat shock transcription factor A2d, hexokinase 2, and SNF1-related protein kinases	[192]
		Starch phosphorylation, chorismate biosynthesis, L-ascorbate biosynthesis and recycling,	[193]

## Data Availability

Not applicable.
